# N^6^-methyladenine is incorporated into mammalian genome by DNA polymerase

**DOI:** 10.1038/s41422-020-0317-6

**Published:** 2020-04-30

**Authors:** Xiaoling Liu, Weiyi Lai, Yao Li, Shaokun Chen, Baodong Liu, Ning Zhang, Jiezhen Mo, Cong Lyu, Jing Zheng, Ya-Rui Du, Guibin Jiang, Guo-Liang Xu, Hailin Wang

**Affiliations:** 1 State Key Laboratory of Environmental Chemistry and Ecotoxicology, Research Center for Eco-Environmental Sciences, Chinese Academy of Sciences, Beijing, 100085 China; 2 University of Chinese Academy of Sciences, Beijing, 100049 China; 3 State Key Laboratory of Molecular Biology, Center for Excellence in Molecular Cell Science/Shanghai Institute of Biochemistry and Cell Biology, Chinese Academy of Sciences, Shanghai, 200031 China; 4 Institute of Environment and Health, Jianghan University, Wuhan, Hubei 430056 China

**Keywords:** DNA methylation, Embryonic stem cells

Dear Editor,

DNA N^6^-methyladenine (6mA), one of the most prevalent epigenetic base modifications in prokaryotes,^1^ is recently found in multicellular eukaryotes.^2–8^ This nucleobase may have epigenetic roles in regulation of retrotransposons, chromatin organization, and so on.^2–8^ However, both our group^9^ and Greer’s group^10^ noticed that eukaryotic DNA is easily contaminated with a minute of bacterial DNA, which carries overwhelmingly abundant 6mA (~2% 6mA/dA).^1^ This brings great challenges for accurate detection of DNA 6mA in eukaryotes in terms of both sample pretreatments and analytical technologies.^9,10^ For example, inconsistent with the report of Wu et al.,^8^ Schiffers et al. failed to detect 6mA above background levels in mouse embryonic stem (mES) cells using sensitive ultra-high-performance liquid chromatography-quadruple mass spectrometry (UHPLC-MS/MS) analysis.^11^ To date, it is of intensive interest to seek conclusive evidence to support the prevalence of this post-replicative adenine modification in mammals.

**Fig. 1:**
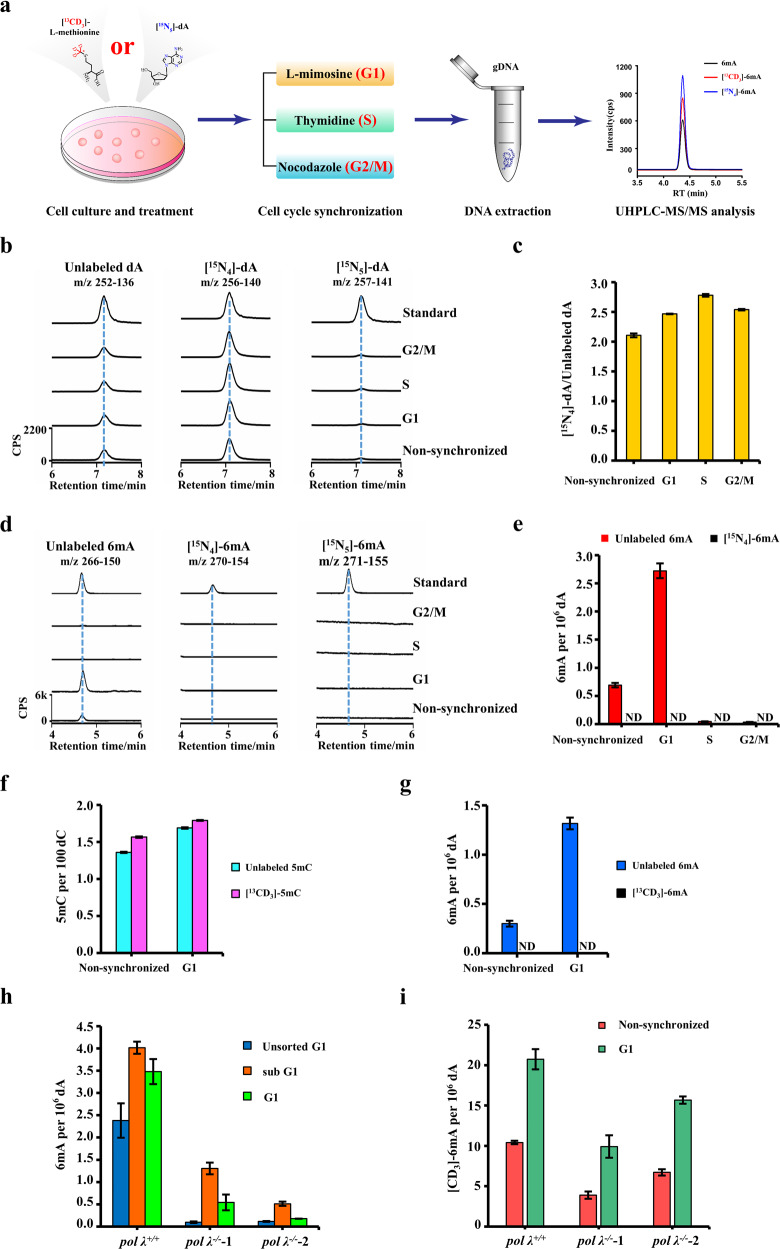
Genomic incorporation of DNA N^6^-methyladenine and the contribution of DNA polymerase λ. **a** Flow diagram of tracing DNA 6mA in mES cells by [^15^N_5_]-dA or [^13^CD_3_]-L-methionine. (**b**, **c**) UHPLC-MS/MS chromatograms (**b**) and quantification (**c**) of unlabeled dA, [^15^N_4_]-dA, and [^15^N_5_]-dA in the genome of mES cells. (**d**, **e**) UHPLC-MS/MS chromatograms (**d**) and quantification (**e**) of unlabeled 6mA, [^15^N_4_]-6mA, and [^15^N_5_]-6mA in the genome of mES cells. Note: [^15^N_5_]-dA was used as an initiation tracer and would be converted into [^15^N_4_]-dA in genomic DNA.^9^ The distinct cell cycle phases are indicated in (**b–e**). (**f**, **g**) UHPLC-MS/MS quantification of [^13^CD_3_]-5mC (**f**) and [^13^CD_3_]-6mA (**g**) in the genomes of non-synchronized and G1 phase ES cells. [^13^CD_3_]-L-methionine was used for tracing stable isotope-labeled methyl group. **h** UHPLC-MS/MS quantification of genomic 6mA levels in *pol λ*^*+/+*^ and *pol λ*^*−/*−^ mES cells. The unsorted cells at G1 phase were directly obtained by L-mimosine treatment. The sorted cells at sub G1 and G1 phases were obtained by flow cytometry sorting of the L-mimosine-treated cells. **i** UHPLC-MS/MS quantification of the labeled genomic 6mA ([CD_3_]-6mA) levels in *pol λ*^*+/+*^ and *pol λ*^−*/*−^ mES cells. The cells were treated with [CD_3_]-m^6^A alone or co-treated with [CD_3_]-m^6^A and L-mimosine. ND, not detected.

These issues prompted us to re-investigate DNA 6mA in mammalian cells. To provide robust and reliable data, a contamination-free UHPLC-MS/MS technology, which is being developed in our lab, was used for ultrasensitive and accurate detection of 6mA in mammals. We measured genomic 6mA in four cultured mammalian cell lines. We observed 6mA in three human cell lines, including HEK293T cells (~0.7 6mA per 10^7^ dA), human mesenchymal stem cells (~2.0 6mA per 10^7^ dA), and human embryonic stem cells (hES cells) (~4.0 6mA per 10^7^ dA). We also detected 6mA in mES cells (~7.0 6mA per 10^7^ dA) (Supplementary information, Fig. S1a, b). As detected by specific PCR analysis, no mycoplasma contamination was observed in all these cultured cells (Supplementary information, Fig. S1c). However, the detected level of 6mA is ~10 folds lower than previously reported.^8^ This might be associated with incomplete release of 6mA from genomic DNA during enzymatic digestion. By spiking 6mA-absent genomic DNA with synthetic 6mA-containing oligonucleotide, we validated a complete release of 6mA by effective enzymatic digestion (Supplementary information, Fig. S1d). On the other hand, the culturing conditions might contribute to the low levels of 6mA in mES cells. Both the study by Schiffers et al. and ours used 2i + LIF, whereas the work by Wu et al.^8^ used a 2i-absent culturing medium. Along this line, it was reported that prolonged Mek1/2 suppression impaired the developmental potential of ES cells.^12^ The replacement of the Mek1/2 inhibitor (PD0325901) with a Src inhibitor (CGP77675) preserved the epigenetic and genomic integrity as well as the developmental potential of mES cells. Hence, five additional culturing conditions were tested (Supplementary information, Fig. S1e). However, similar levels of DNA 6mA were detected (4.0–8.0 6mA per 10^7^ dA; Supplementary information, Fig. S1f). By treatment of late G1-phase arresting agent L-mimosine (Fig. 1a), interestingly, we observed an accumulation of genomic 6mA in both mouse (2.7 6mA per 10^6^ dA, Fig. 1d, e) and human ES cells (1.9 6mA per 10^6^ dA, Supplementary information, Fig. S2a). We also observed an increase of 6mA in HEK293T cells (Supplementary information, Fig. S2b). By using early G1-phase arresting palbociclib, we observed a moderate increase of 6mA (Supplementary information, Fig. S2c). For the first time, we reported the accumulation of 6mA in G1 phase.

Next, we exploited unique stable isotope-labeled deoxyadenosine tracing technology to investigate the 6mA origin. Previously, we showed that [^15^N_5_]-2'-deoxyadenosine ([^15^N_5_]-dA) tracer can be incorporated into genomic DNA in the form of [^15^N_4_]-dA.^9^ If there is any methyltransferase-dependent 6mA in any cultured cells, [^15^N_4_]-6mA should be detected. Another advantage of this [^15^N_5_]-dA tracing technology is its capacity of discriminating prototype bacterial mycoplasmas, which mainly carry [^15^N_5_]-6mA but not [^15^N_4_]-6mA, from host cells.^9^ As revealed by UHPLC-MS/MS analysis, >67% of genomic dA was labeled in the form of [^15^N_4_]-dA (Fig. 1b, c). dG was also efficiently labeled (Supplementary information, Fig. S3a, b). These results are consistent with our recent work.^9^ Despite efficient labeling of dA, we failed to detect any [^15^N_4_]-6mA in three tested cell lines, including mES cells (Fig. 1d, e), hES cells and HEK293T cells (Supplementary information, Fig. S2), at all cell cycle phases. Moreover, under six culturing conditions (Supplementary information, Fig. S1e), we did not detect any [^15^N_4_]-6mA in the genomes of mES cells. Of note, our assay can detect ten labeled 6mA from one human genome. In contrast, we observed [^15^N_4_]-6mA by transfecting HEK293T cells with a plasmid carrying an *E. coli* 6mA methylase *dam* mutant gene (data not shown), which has an activity of 100 times lower than wild type.

To corroborate our results, we used second stable isotope-labeling reagent [^13^CD_3_] L-methionine. This chemical can be in vivo converted into stable isotope-labeled methyl donor S-adenosyl-L-methionine, which must be utilized by possible 6mA methylases to generate DNA N^6^-methylated adenine. If there was any methyltransferase to act on N^6^ atom of dA, we could detect the formed [^13^CD_3_]-6mA. Consistent with the above results, we did not detect any [^13^CD_3_]-6mA in the treated mES cells at all cell cycle phases (Fig. 1g and Supplementary information, Fig. S4b). In contrast, we did observe [^13^CD_3_]-5mC in mES cells ([^13^CD_3_]-5mC/total 5mC: >50%) (Fig. 1f and Supplementary information, Fig. S4a).

Collectively, all the above results supported that the observed 6mA is independent of the methylases, proving the origin of methylase-independent 6mA.

In view of a lack of methylase-generated 6mA, we proposed that the observed 6mA is caused by DNA polymerase-dependent incorporation. If so, the tested cells should have an ability to incorporate 6mA into their genomes. Indeed, by treating mES cells with deoxyribonucleoside N^6^-methyldeoxyadenosine (50–800 μM) (Supplementary information, Fig. S5a, b), we observed dose-dependent incorporation of 6mA into the genome (0.9–3.5 6mA per 10^6^ dA). Similarly, by treatment of ribonucleoside N^6^-methyladenosine (m^6^A, 10–50 μM), we also observed a dose-dependent increase in genomic 6mA (4.2–7.1 6mA per 10^6^ dA) (Supplementary information, Fig. S5c, d). Moreover, the treatment of N^6^-methyladenine base-containing DNA or RNA fragments also increased the level of genomic 6mA in mES cells (Supplementary information, Fig. S5e, f). In contrast, the treatment of N^6^-methyladenine base-absent DNA or RNA fragments could not induce any 6mA increase (Supplementary information, Fig. S5e, f).

To further provide direct evidence on the incoporation of 6mA into mammalian genome, we used two stable isotope-labeled N^6^-methyladenines (Supplementary information, Fig. S6a, b): 2'-deoxyribonucloside [^15^N_5_]-6mA and ribonucleoside [CD_3_]-m^6^A. Both treatments could induce a dramatic increase in genomic 6mA in the respective labeled forms (Supplementary information, Fig. S6c–e).

Now the question is how the genome of mES cells incorporates 6mA. To answer this question, we first explored a routinely used high-fidelity Taq DNA polymerase as a substitute for the template-dependent, high-fidelity replication polymerases in mammalian cells. By replacing 2′-deoxyadenosine triphosphate (dATP) with N^6^-methyl-dATP (N^6^mdATP), we indeed observed the incorporation of 6mA in PCR products (Supplementary information, Fig. S7a, b). However, when a mixture of N^6^mdATP and dATP with a ratio of 1:1000 was used for PCR amplification, we could not observe any 6mA incorporation (Supplementary information, Fig. S7b). These results suggest that high-fidelity polymerases prefer using dATP rather than N^6^mdATP for DNA synthesis.

Next, we turned our attention to one of the template-independent X family DNA polymerases, Pol λ (lambda). Compared to non-synchronized cells, the mRNA expression of Pol λ increased in late G1 phase mES cells (Supplementary information, Fig. S8a). Pol λ knockdown effectively reduced both its mRNA expression and the 6mA abundance in non-synchronized and late G1 phase cells (Supplementary information, Fig. S8b, c). We further generated two *Pol λ*-knockout mES cell lines (Supplementary information, Fig. S8d, e). We treated the cells with L-mimosine to obtain G1 phase-dominant cells, and observed that the abolishment of *pol λ* reduced the 6mA abundance (Fig. 1h, indicated as the unsorted G1). Moreover, we sorted out both sub G1 phase and G1 phase L-mimosine-treated mES cells (Supplementary information, Fig. S9), and found that genomic 6mA accumulated at both sub G1 and G1 phases (Fig. 1h). The depletion of Pol λ caused a dramatic 6mA decrease in both G1 phase and sub G1 phase. Interestingly, Pol λ depletion also impaired the 6mA incorporation capacity of mES cells as revealed by the treatment of extracellular [CD_3_]-m^6^A (Fig. 1i).

Noteworthy, as indicated by an increase in the cell number of apoptosis-related sub G1 phase, L-mimosine treatment also increased apoptosis of pol λ-knockout mES cells (Supplementary information, Fig. S9a–d). Since Pol λ participates in non-homologous end joining (NHEJ) repair^13^ via its BRCT domain and NHEJ accounts for a large proportion of DNA repair in the G1 phase,^14^ Pol λ may incorporate 6mA into the genome through NHEJ repair pathway. This may implicate a potential association of 6mA with NHEJ.

To further investigate the origin of DNA 6mA in mES cells, we knocked out a potential methylase gene *mettl4*^15^ and a demethylase candidate gene *alkbh1*^8^ by CRISPR/Cas9 technology. We obtained two *mettl4*^*−/−*^ mESC strains (Supplementary information, Fig. S10a, b), five *alkbh1*^*−/−*^ mESC strains (Supplementary information, Fig. S11a, b, e) and one *alkbh1*^*−/−*^ HEK293T strain (Supplementary information, Fig. S11c, d, f). Evidently, *mettl4* knockout could not reduce  the level of genomic 6mA in mES cells (Supplementary information, Fig. S10c). By knockout of *alkbh1* gene in mES cells, interestingly, we could see a 6mA decrease in two strains, but a 6mA increase in three strains (Supplementary information, Fig. S11g). By knockout of *alkbh1* gene in HEK293T cells, we only observed a 6mA decrease (Supplementary information, Fig. S11h). The observation of contrary trends on genomic 6mA abundance in *alkbh1*^*−/−*^ strains should reflect a varying adaption of the mES cells to *alkbh1* knockout rather than elimination of a demethylation function. Of note, even accompanying with the increase in 6mA for three *alkbh1*^*−/−*^ strains (Supplementary information, Fig. S11g), we did not see any stable isotope-labeled 6mA (data not shown). This observation confirmed the absence of methylase-generated DNA 6mA in cultured mES cells. Moreover, by the treatment of [CD_3_]-m6A, we observed genomic incorporation of [CD_3_]-6mA in both mES cells and HEK293T cells (Supplementary information, Fig. S11i, j). However, *Alkbh1* knockout could not increase the level of [CD_3_]-6mA in both cells (Supplementary information, Fig. S11i, j). Collectively, these results did not support that Alkbh1 plays a role in the elimination of genomically incorporated 6mA.

In this study, we exploited two stable isotope-labeling strategies for indicating either nucleobase adenine (by ([^15^N_5_]-dA) or N^6^-methyl group (by [^13^CD_3_]-L-methionine) to trace methylase-generated genomic 6mA in mammalian ES cells. Adenine methylation would yield genomic 6mA in a form bearing a stable isotope label. Astonishingly, by both labeling strategies, the detected 6mA is present exclusively in the non-labeled form in both human and murine ES cells. We also found that non-labeled genomic 6mA increases with extracellular N^6^-methyladenine base-containing (deoxy)ribonucleosides and RNA or DNA fragments. By the use of either deoxyribonucleoside [^15^N_5_]-6mA or ribonucleoside [CD_3_]-m^6^A as a stable isotope tracer, strikingly, we observed genomic DNA 6mA predominantly in the stable isotope-labeled form. These data consistently support thepresence of DNA polymerase-dependent incorporation of 6mA and the absence of methylase-generated 6mA at least in the tested cells. DNA polymerase λ is identified as one of major polymerases responsible for 6mA accumulation in late G1 phase. Noteworthy, the incorporated DNA 6mA is not altered by depletion of demethylase candidate Alkbh1 or methylase candidate Mettl4. Overall, our data suggest a new origin of DNA N^6^-methyladenine base in mammals, implicating the complexity of this rare base in the context of genome function.

### Supplementary information

Supplementary information, Materials and Figures

Supplementary information, Tables
