# Supplementary material for: N6-methyladenine is incorporated into mammalian genome by DNA polymerase
Source: Cell Res. 2020 Apr 30;31(1):94–7. doi: 10.1038/s41422-020-0317-6 (PMC7853133; doi:10.1038/s41422-020-0317-6)
Supplement: Supplementary file 1 — Supplementary information, Materials and Figures [file 41422_2020_317_MOESM1_ESM.pdf]

Supplementary information

N^6-methyladenine is incorporated into mammalian genome by DNA polymerase

Xiaoling Liu[1], Weiyi Lai[1], Yao Li[1,2], Shaokun Chen[2], Baodong Liu[1], Ning Zhang [1], Jiezhen Mo[1,2], Cong Lyu[1,2], Jing Zheng[1,2], Ya-Rui Du[4], Guibin Jiang[1], Guo-Liang Xu[4], Hailin Wang[1,2,3]

[1]State Key Laboratory of Environmental Chemistry and Ecotoxicology, Research Center for Eco-Environmental Sciences, Chinese Academy of Sciences, Beijing 100085, China;
[2]University of Chinese Academy of Sciences, Beijing 100049, China;
[3]State Key Laboratory of Molecular Biology, Institute of Biochemistry and Cell Biology, Shanghai Institutes for Biological Sciences, Chinese Academy of Sciences, Shanghai 200031, China;
[4]Institute of Environment and Health, Jianghan University, Wuhan 430056, China

These authors contributed equally: Xiaoling Liu, Weiyi Lai, Yao Li,
Correspondence: Hailin Wang (hlwang@rcees.ac.cn)

Materials and methods

Chemicals and materials.

Deoxyribonuclease I (DNase I) and calf intestinal alkaline phosphatase (CIP) were obtained from New England Biolabs (MA, USA). Crotalus adamanteus venom phosphodiesterase I (SVP) was purchased from Worthington Biochemical Corporation (Lakewood, USA). Ultrapure water was prepared using ELGA PureLab-Ultra purifier (High Wycombe, UK). Glycogen (R0561) was ordered from Thermo Fisher Scientific (MA, USA). [$^{15}N_5$]-dA (purity: 96-98%) was purchased from Cambridge Isotope Laboratories (MA, USA). 2′-Deoxyadenosine monohydrate (dA·H2O, ≥ 99%), 2′-deoxyguanosine monohydrate (dG·H2O, ≥ 99%), 2′-deoxycytidine (dC, ≥ 99%), Gelatin, L-mimosine, thymidine, nocodazole, and formic acid (for mass spectrometry, ≈ 98%) were ordered from Sigma-Aldrich (MO, USA). Methanol of LC-MS grade (purity: >99.9%) were ordered from Honeywell (Morristown, NJ, USA). N^6-methyldeoxyadenosine (6mA) was ordered from Santa cruz Biotechnology, Inc. (Texas, USA). [$^{13}CD_3$] L-methionine was ordered form TriLink BioTechnologies (CA, USA). [CD_3]-m^6A was purchased from Toronto Research Chemicals (North York, Canada). [$^{15}N_5$]-6mA was synthesized and purified in our own lab. dCTP, dTTP, dGTP, and dATP were ordered from New England Biolabs (MA, USA) and N6-methyl-dATP (N6mdATP) were from Fermentas (MA, USA).

Cell culture

Mouse embryonic stem cells (mES cells, 129 SvEv) were seeded at 1:10 in 0.1% sterilized gelatin coated dishes (6 cm) and cultured in DMEM high glucose medium (Thermo Fisher Scientific, MA, USA), supplemented with 0.1 mM nonessential amino acids (Gibco, NY, USA), 2.0 mM L-Glutamine (Gibco, NY, USA), 0.1 mM β-mercaptoethanol, 1.0 mM sodium pyruvate (Gibico, NY, USA), 1000 U/mL leukemia inhibitor factor (LIF) (Millipore, Billerica, MA, USA), 1.0 μM PD0325901 (Stemgent, Cambridge, MA, USA), 3.0 μM CHIR99021 (Stemgent, Cambridge, MA, USA), and 20% fetal bovine serum (FBS) (Corning, NY, USA).

In addition, two types of media were also used for culturing mES cells. 1) Serum plus LIF medium (S/L medium): DMEM high glucose medium, supplemented with 0.1 mM nonessential amino acids, 2 mM L-Glutamine, 0.1 mM β-mercaptoethanol, and 1000 U/mL LIF and 1× penicillin–streptomycin (Invitrogen). 2) N2B27 medium: 1:1 mixture of DMEM/F12 (Thermo Fisher Scientific), supplemented with N2 (Thermo Fisher Scientific) and Neurobasal media (Thermo Fisher Scientific) supplemented with B27 (Thermo Fisher Scientific), 1000 U LIF, and 1× penicillin–streptomycin.

Human embryonic stem (hES) cell line H1 (feeder-free) was ordered from Stem Cell Bank, Chinese Academy of Sciences. The hES cells were grown in the matrigel (BD Biosciences, New Jersey, USA) coated dishes and cultured with mTeSR1 Complete Kit (SCSP-662, Chinese Academy of Sciences).

Human bone marrow-derived mesenchymal stem cells (hMSC) were cultured in a laboratory-prepared medium. The preparation protocol described as follows: 485 mL of mesenchymal stem cell basal medium (ATCC, VA, USA), supplemented with 35 mL of FBS (Gibco, NY, USA), 125 pg/mL rhFGF basic (233-FB-025, R&D systems, Minnesota, USA), 15 ng/mL rhIGF-1 (291-G1-200, R&D systems, Minnesota, USA), 2.4 mM L-Alanyl-L-Glutamine (A8185-5G, MO, USA), 100 U/mL penicillin, and 100 μg/mL streptomycin (Corning).

Human embryonic kidney cell line HEK293T was cultured in DMEM (Hyclone, Thermo Fisher Scientific Inc., MA, USA) medium supplemented with 10% FBS (Corning), 100 U/mL penicillin, and 100 μg/mL streptomycin (Corning).

Of note, all the cell lines were cultured in a humidified incubator at 37 °C supplemented with 5% CO_2.

The treatment of cultured cells with (deoxy)ribonucleoside [$^{15}N_5$]-dA, m^{6}A, 6mA, [$^{15}N_5$]-6mA or [$^{13}CD_3$]-L- Methionine

1.0×10^5 mES cells were seeded in 6-well cell culture cluster (Corning), and cultured in 2.0 ml medium. The final concentration of treated (deoxy)ribonucleosides was used as stated, and the final concentration of treated [$^{13}CD_3$]-L-methionine was 30 μg/mL. The cells were harvested for DNA extraction and UHPLC-MS/MS analysis after 4 days' treatment, or as stated.

Synchronization of cultured cells

The heavy stable isotope-labeled cells were synchronized by specific cell cycle inhibitors. Briefly, mES cells were arrested in G1, S, and G2/M phases by 16 h treatment of 400 μM mimosine or 100 nM palbociclib, 2.0 mM thymidine, and 0.4

μg/mL nocodazole, respectively.

Transient transfection of 6mA-DNA oligo or m^{6}A-RNA oligo and DNA extraction
1.0 × 10^5 mES cells were seeded into a six-well plate and cultured for 24 h before transfection using Lipofectamine RNAiMAX (Life Technologies) according to the manufacturer's instructions with minor modifications. 20 μL RNAiMAX reagent and 4 nmol of oligos were used and incubated with cells for 48 h. The oligos were purchased from Takara Bio (Dalian, China) and the sequences were listed in Supplementary information, Table S1.

To remove possible co-extracted oligo, ammonium acetate precipitation was performed. The genomic DNA that was purified by Promega Wizard genomic DNA purification kit was dissolved in 20 mM tris-HCl buffer (pH 8.0), then mixed with an equal volume of 5.0 M ammonium acetate (pH 5.2), and then added with three volume of ethanol and vortexed. Next, centrifuged by 12,000 rpm for 2 min at room temperature. The DNA pellet was washed with ethanol/water (7:3, v/v) three times, and dried in air. The dried DNA was then re-dissolved in ddH_2O. The concentration of the purified DNA was determined with NanoDrop 2000 (Thermo Fisher Scientific, MA, USA) and the quality was evaluated with the ratio of absorbance at 260 nm and 280 nm.

SiRNA transient transfection followed by Synchronization
1.0 × 10^5 mES cells were seeded into a six-well plate and grew for 24 h. Then, the cells were transfected with 25 pmol siRNA (GenePharma, Suzhou, China) using Lipofectamine RNAiMAX (Life Technologies) following the manufacturer's guidelines and incubated for 30 h. Afterwards, mES cells were arrested at the G1 phase by the treatment of 400 μM mimosine for 16 h. The mRNA level of pol λ was measured by quantitative real time-PCR. The cells were harvested for DNA extraction and RNA extraction, respectively. The sequence of siRNAs used in this study were listed in Supplementary information, Table S2.

mRNA expression analysis
Total RNA was extracted from cells by TRIzol reagent (Life technologies Corporation). 1.0 μg RNA was reversely transcribed to cDNA with Promega reverse transcription system. Real-time PCR was performed using Promega GoTaq qPCR Master Mix following the manufacturer's protocol on Stratagene Mx3005P real-time PCR System (Agilent Technologies, CA, USA). The pol λ mRNA levels were normalized by the Gapdh mRNA levels. The RT-qPCR primer sequences were listed in Supplementary information, Table S3.

Generation of knockout cell lines by CRISPR/Cas9
Guide RNA oligos were annealed and cloned into a PX458 plasmid (Addgene plasmid #48138). mES cells were first seeded into a six-well plate at low density and grew for 12 h. Then the cells were transfected with 5.0 μg of the PX458-sgRNA plasmid. The transfection was performed using Lipofectamine 2000 (Life Technologies) according to the manufacturer's instructions. The transfected cells were further incubated for 24

h. Afterwards, the mES cells were digested into individual cells and sorted into 96-well plates according to GFP signal by BD Ariall flow cytometer (BD Biosciences, New Jersey, USA). Then, after three-days culturing, ~40 mES cell colonies were picked up for PCR screening. The PCR products were verified by T cloning vector sequencing using pClone007 Blunt Vector Kit following instructions (TsingKe biological technology, Beijing, China).

The sgRNA sequence and the sequence of PCR primers flanking guide RNA were listed in Supplementary information, Table S4.

General protocol for genomic DNA extraction and enzymatic digestion

The adherent cells were gently washed twice with 1 × phosphate-buffered saline (PBS buffer, 8.1 mM Na2HPO4; 1.76 mM KH2PO4; 136.89 mM NaCl; 2.67 mM KCl), and detached by 1.0 min trypsin digestion. The genomic DNA was extracted according to the instructions of the Promega wizard® genomic DNA purification kit (Promega, WI, USA). The concentration of the extracted DNA fractions was determined with NanoDrop 2000 (Thermo Fisher Scientific, MA, USA) and the DNA quality was evaluated with the ratio of absorbance at 260 nm and 280 nm.

To obtain deoxyribonucleosides, per 5.0 μg DNA was digested with 0.5 U CIP (New England Biolabs), 0.25 U DNase I (New England Biolabs), and 0.25 U SVP (Worthington) at 37 °C overnight. To remove the digestive enzymes, DNA solution was ultrafiltered with ultrafiltration tubes (molecule weight cutoff: 3 KDa; Pall Corporation, Port Washington, NY, USA). The ultrafiltered solution was then subjected to UHPLC - MS/MS analysis.

UHPLC-MS/MS analysis

The UHPLC-MS/MS analysis was performed on an Agilent 1290 II UHPLC system coupled with an ESI-triple quadrupole mass spectrometer (6470, Agilent Technologies, CA, USA). A ZORBAX SB-Aq column (2.1 × 100 mm, 1.8 μm particle size, Agilent) was employed for the separation of deoxyribonucleosides.

The mass spectrometer was operated under positive ionization using multiple reactions monitoring (MRM) mode. The selective MRM transitions were monitored as follows: m/z 266→150 for 6mA, m/z 269→153 for [CD_3]-6mA, m/z 270→154 for [$^{15}N_4$]-6mA or [$^{13}CD_3$]-6mA, m/z 271→151 for [$^{15}N_5$]-6mA, m/z 242→126 for 5mC, m/z 245-129 for [D_3]-5mC, m/z 246→130 for [$^{13}CD_3$]-5mC, m/z 252→136 for dA, m/z 256→140 for [$^{15}N_4$]-dA, m/z 257→141 for [$^{15}N_5$]-dA, m/z 268→152 for dG and m/z 272→156 for [$^{15}N_4$]-dG. The fragmentation voltage for all the MRM transitions were set at 90 V to allow efficient transit of precursor ions. Nitrogen gas was used for nebulization and desolvation. The nebulization gas pressure was set at 40 psi, and the temperature and the flow rate of desolvation gas were set at 300 °C and 9.0 L/min, respectively. High purity nitrogen (99.999%) was used as collision gas.

***In vitro* incorporation of 6mA in replicated DNA by high fidelity Taq DNA polymerase.**

To explore whether DNA polymerase can incorporate 6mA during DNA synthesis or

replication *in vitro*, we performed a 6mA-involved polymerase chain reaction (PCR). 100 ng genomic DNA of mES cells was used as the template, and mixed with 0.5 μM of each primer and 5 μL HiFiTaq PCR buffer, 5 U HiFiTaq DNA polymerase (GeneStar, Shanghai, China), dCTP, dTTP, dGTP, and dATP or N6mdATP (50 μM each) in a total volume of 50 μL. The PCR products were analyzed by 1% agarose gel electrophoresis. The PCR products separated by the agarose gel electrophoresis were extracted from the gel using a PCR clean up kit (Promega, WI, USA), and the extracted DNA was analyzed for detection of incorporated 6mA by UHPLC-MS/MS.

Flow cytometry sorting of mES cells at the G1 and subG1 phase.

The mES cells were arrested at the G1 phase by a 16 h treatment of 400 μM mimosine, and then trypsinized to be dispersed into single-cell suspensions. The dispersed cells were washed with 1 × PBS and collected by centrifugation. Re-suspended the collected cells in pre-chilled PBS, discarded the supernatant, and slowly dropwisely added 3.0 mL of pre-chilled absolute ethanol (final concentration: 75% v/v), then rested the cells overnight at 4 °C. Next, washed the fixed cells with pre-chilled PBS twice, removed PBS by centrifuge at 1000 rpm for 5 minutes, and added 200 μL of RNase A (50-100 μg/mL) and incubated at 37 °C for 30 min, then discarded the supernatant, and added 500 μL ethidium bromide (PI, 50 μg/mL) and incubated at 4 °C for 30 min in the dark. Finally, filtered the mES cells through a 200-mesh strainer and sorted the cells within 1 h following PI staining by a BD Ariall flow cytometer (BD Biosciences, NJ, USA). The flow cytometer was controlled using BD FACSDiva software. The sorted cells were collected by centrifugation, and genomic DNA was extracted for UHPLC-MS/MS analysis.

Western blotting analysis

The expression of *Alkbh1* gene in *Alkbh1* knock out cells was analyzed by western blot. Briefly, the total protein of wild type or *Alkbh1*$^{-/-}$ cells was extracted by cold RIPA lysis buffer (Beyotime) with 1% PMSF, and then separated by NuPAGE 4-12% bis-tris gel in MOPS SDS running buffer (Invitrogen, California, USA), and then electrophoretically transferred onto 0.45 μm PVDF membrane (Millipore, Darmstadt, Germany). After blocking with 5% skim milk powder in TBST (20 mM Tris, pH 7.4, 150 mM NaCl, 0.1% Tween-20) for 1 h, the membrane was then incubated with a primary antibody (ab195376, Abcam, Cambridge, UK) diluted at 1:1000 in 5% milk at 4 °C overnight. Subsequently, the membranes were incubated with secondary antibody (goat anti-rabbit IgG, BS10007, Bioworld, MN, USA) for 1 h at room temperature. Then, the blot was imaged by Odyssey CLX infrared imaging system (LI-COR Biotechnology, Lincoln, NE, USA)

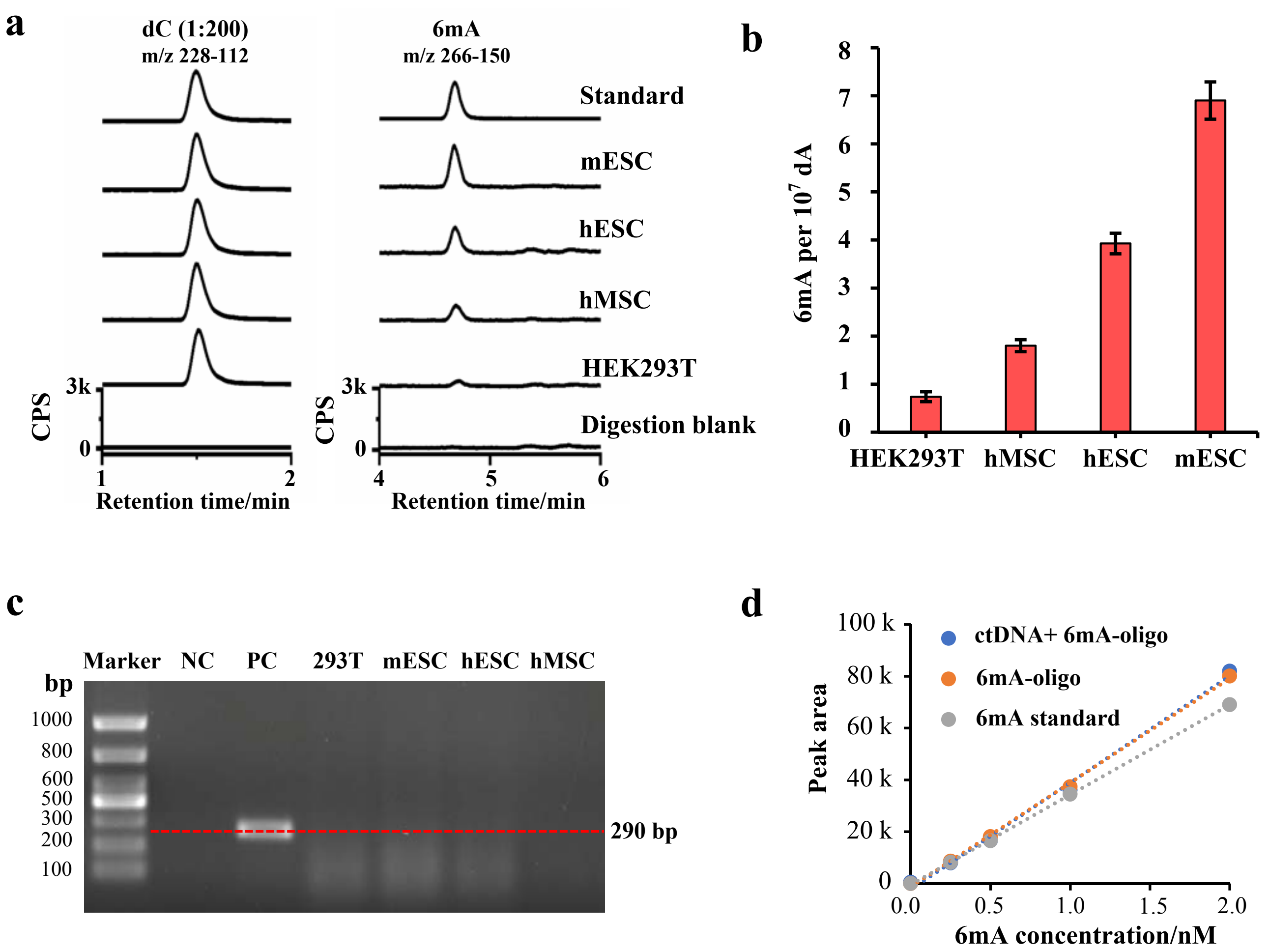

e

No.	1	2	3	4	5	6
Medium	S/L	S/L	S/L	S/L	N2B27	N2B27
1 mM sodium pyruvate	+	-	+	+	-	-
3 µM CHIR99021	+	-	+	+	+	+
1 µM PD0325901	+	-	-	-	+	-
1.5 µM CGP77675	-	-	-	+	-	+

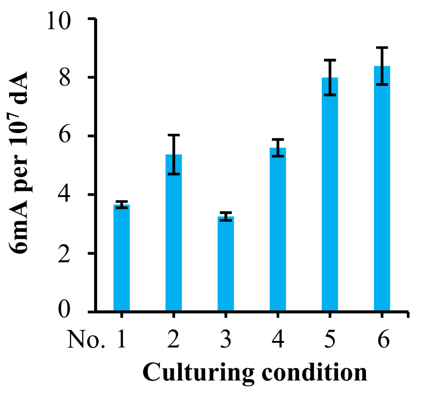

Fig. S1 UHPLC-MS/MS quantification of genomic 6mA levels in four mammalian cell lines. (**a, b**) UHPLC-MS/MS chromatograms (**a**) and quantification (**b**) of DNA 6mA in four cell lines (mESC, hESC, hMSC, and HEK293T). The peaks of DNA 6mA were normalized against the peaks of dC. Error bar: s.d. n = 3. (**c**) Agarose gel electrophoresis analysis of the PCR products (290 bp) for the detection of mycoplasma in four cell lines. NC: negative control; PC: positive control. Agarose gel: 1% agarose. (**d**) Release of 6mA from 6mA-oligo alone or the spiked solution of 6mA-oligo and 6mA-absent calf thymus DNA (ctDNA). The released 6mA was evaluated by UHPLC-MS/MS analysis. X-axis indicates the concentration of 6mA in the 6mA-oligo solution or the ctDNA + 6mA-oligo solution. Y-axis indicates the peak area (dimensionless) of the released or standard 6mA. (**e**) Six conditions for culturing mES cells. The details about the medium ingradients see Materials and methods in Supplementary information. (**f**) UHPLC-MS/MS quantification of DNA 6mA levels in mES cells that were cultured under six different conditions (see **e**). Error bar: s.d. n = 3.

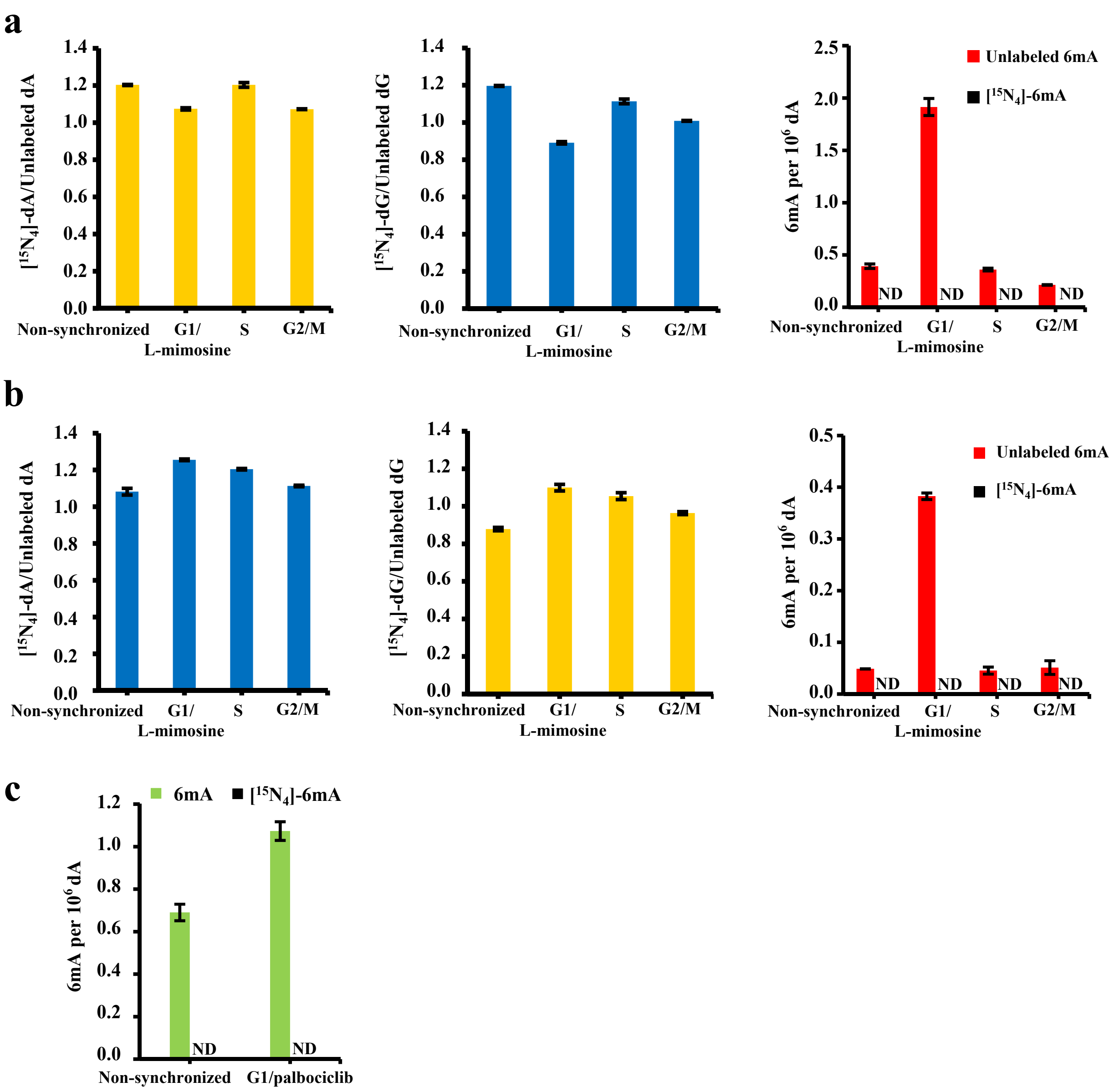

Fig. S2 The levels of the unlabeled and labeled 6mA in genomic DNA.
The cultured cells were co-treated with initial stable isotope tracer [$^{15}N_5$]-dA and one cell-cycle-arrested chemical as described in Supplementary information, Materials and Methods. (**a**) The labeling efficiency of [$^{15}N_4$]-dA and [$^{15}N_4$]-dG and the 6mA levels in the genomic DNA of human ES cells. (**b**) The labeling efficiency of [$^{15}N_4$]-dA and [$^{15}N_4$]-dG and the 6mA levels in the genomic DNA of HEK293T cells. (**c**) The 6mA levels in the genomic DNA of palbociclib-treated mES cells. Error bar: s.d. n = 3. ND: not detected.
Note: L-mimosine and palbociclib arrest the cells at G1 and late G1 phases, respectively.

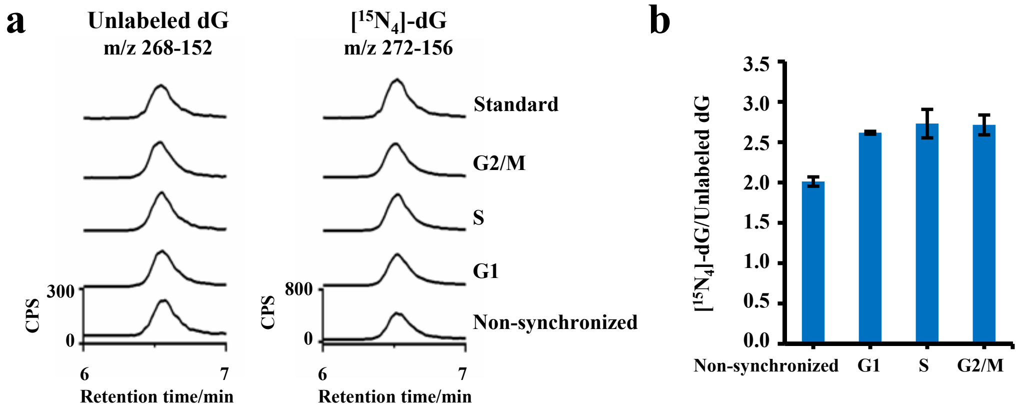

Fig. S3 Labeling efficiency of [$^{15}N_4$]-dG in genomic DNA of mES cells at distinct cell cycle phases.

The mES cells were treated with initial stable isotope tracer [$^{15}N_5$]-dA. Cell-cycle-arrested chemicals were used as described in Supplementary information, Materials and Methods. (**a**) UHPLC-MS/MS chromatograms of [$^{15}N_4$]-dG. The peaks of [$^{15}N_4$]-dG were normalized against unlabeled dG. (**b**) The labeling ratio of [$^{15}N_4$]-dG to the unlabeled dG. Error bar: s.d. n = 3.

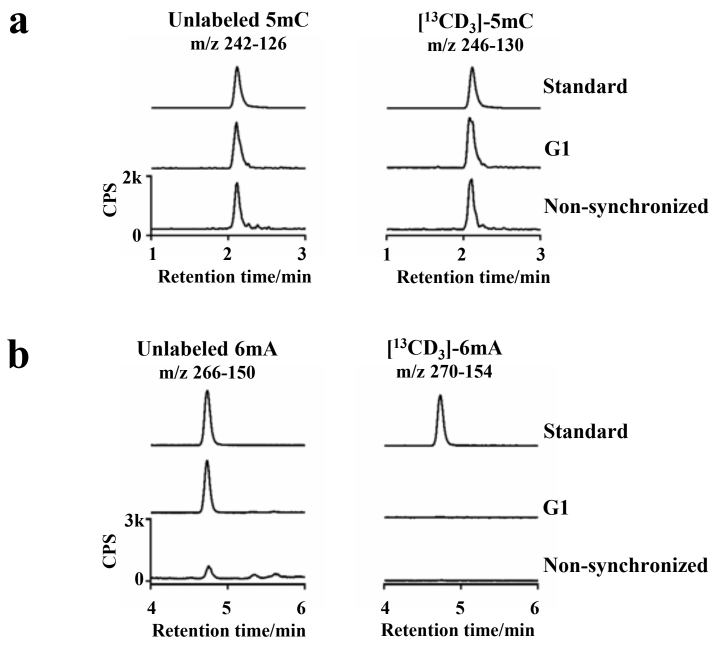

Fig. S4 Identification of methylase-independent DNA N6-methyladenine (6mA) by a stable isotope tracer [$^{13}CD_3$]-L-methionine.

(**a**) UHPLC-MS/MS chromatograms of 5mC and [$^{13}CD_3$]-5mC in non-synchronized and G1 phase-arrested mES cells. (**b**) UHPLC-MS/MS chromatograms of the unlabeled 6mA and [$^{13}CD_3$]-6mA in non-synchronized and G1 phase-arrested mES cells.

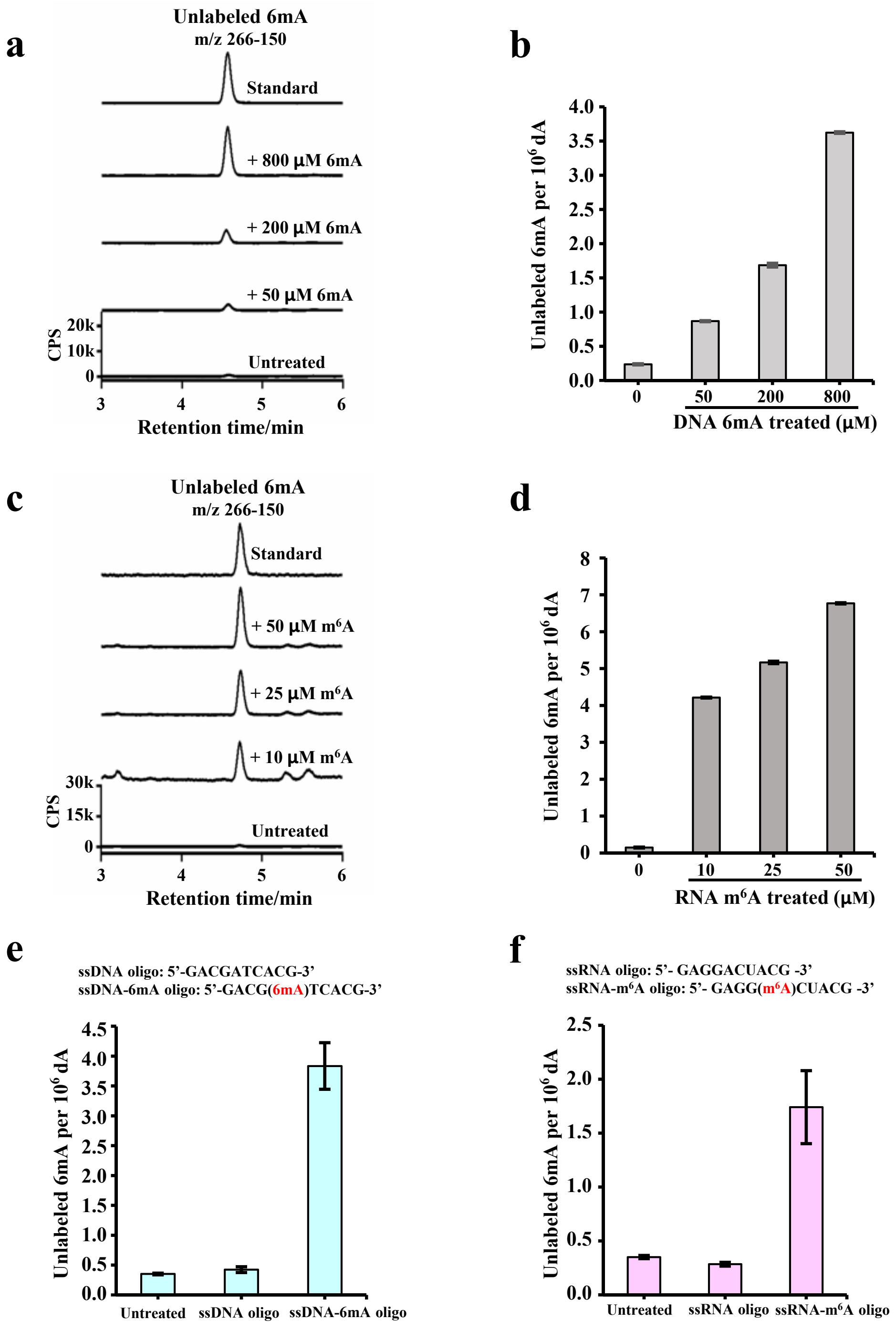

Fig. S5 Genomic incorporation of 6mA in mES cells by the treatment of exogenous N6-methyladenine base-containing (deoxy) ribonucleosides.

(**a-d**) Dose-dependent increase in genomic 6mA by the treatment of the deoxyribonucleoside 6mA (**a, b**) and the ribonucleoside m^6A (**c, d**) in mES cells. (**a, c**) UHPLC-MS/MS chromatograms; (**b, d**) Quantification of DNA 6mA in mES cells. (**e, f**) Genomic incorporation of 6mA in mES cells by the treatment of synthetic DNA-6mA oligo (**e**) or RNA-m^6A oligo (**f**). Error bar: s.d. n = 3.

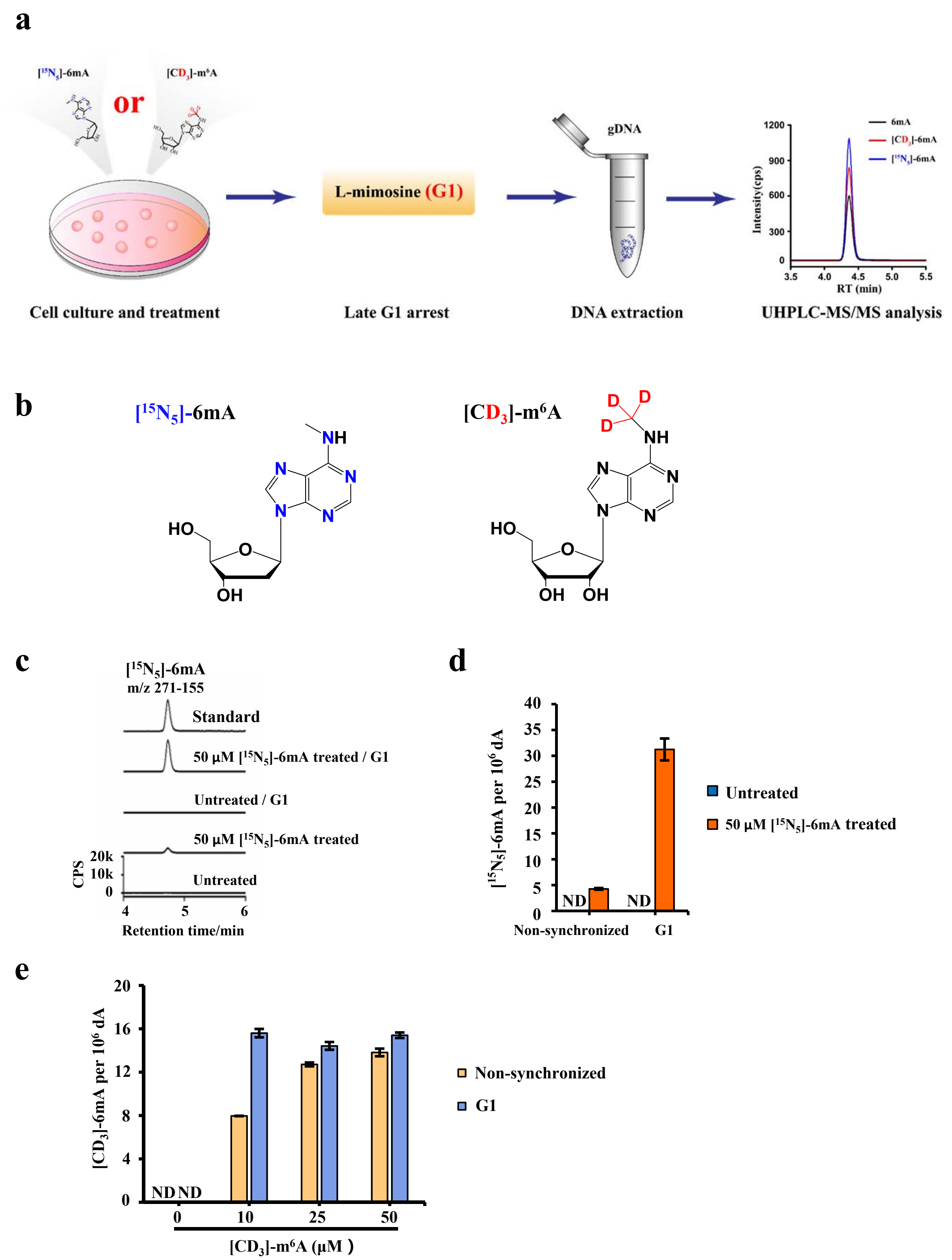

Fig. S6 Genomic incorporation of 6mA by the treatment of stable isotope-labeled deoxyribonucleoside 6mA and ribonucleoside m^{6}A in mES cells.

(**a**) Flow diagram of stable isotope labeling for tracing genomic incorporation. (**b**) Chemical structures of stable isotope tracers, [$^{15}N_5$]-6mA and [CD_3]-m^{6}A. The heavy stable isotope-carrying atoms were highlighted in blue (^{15}N) or red (D). (**c, d**) UHPLC-MS/MS chromatograms (**c**) and quantification (**d**) of genomic [$^{15}N_5$]-6mA in non-synchronized and G1 phase-arrested mES cells. The cells were treated with [$^{15}N_5$]-6mA. (**e**) Quantification of genomic [CD_3]-6mA in non-synchronized and G1 phase-arrested mES cells. The cells were treated with [CD_3]-m^{6}A. ND: not detected. Error bar: s.d. n = 3.

a

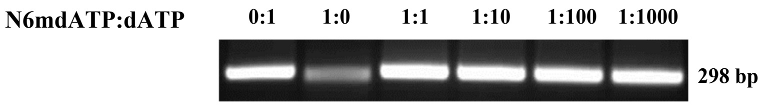

b

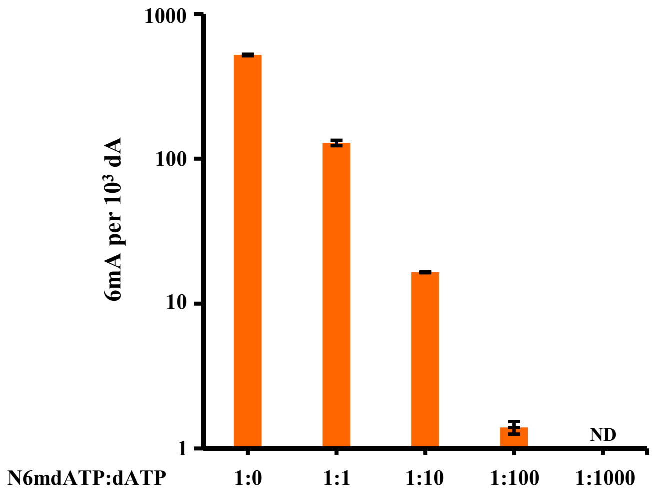

Fig. S7 In vitro incorporation of 6mA in replicated DNA by high fidelity Taq DNA polymerase.
(**a**) PCR products that were separated by 1% agarose gel electrophoresis and stained with GelRed. (**b**) UHPLC-MS/MS quantification of the 6mA levels of PCR products. ND: not detected. Error bar: s.d. n = 3.

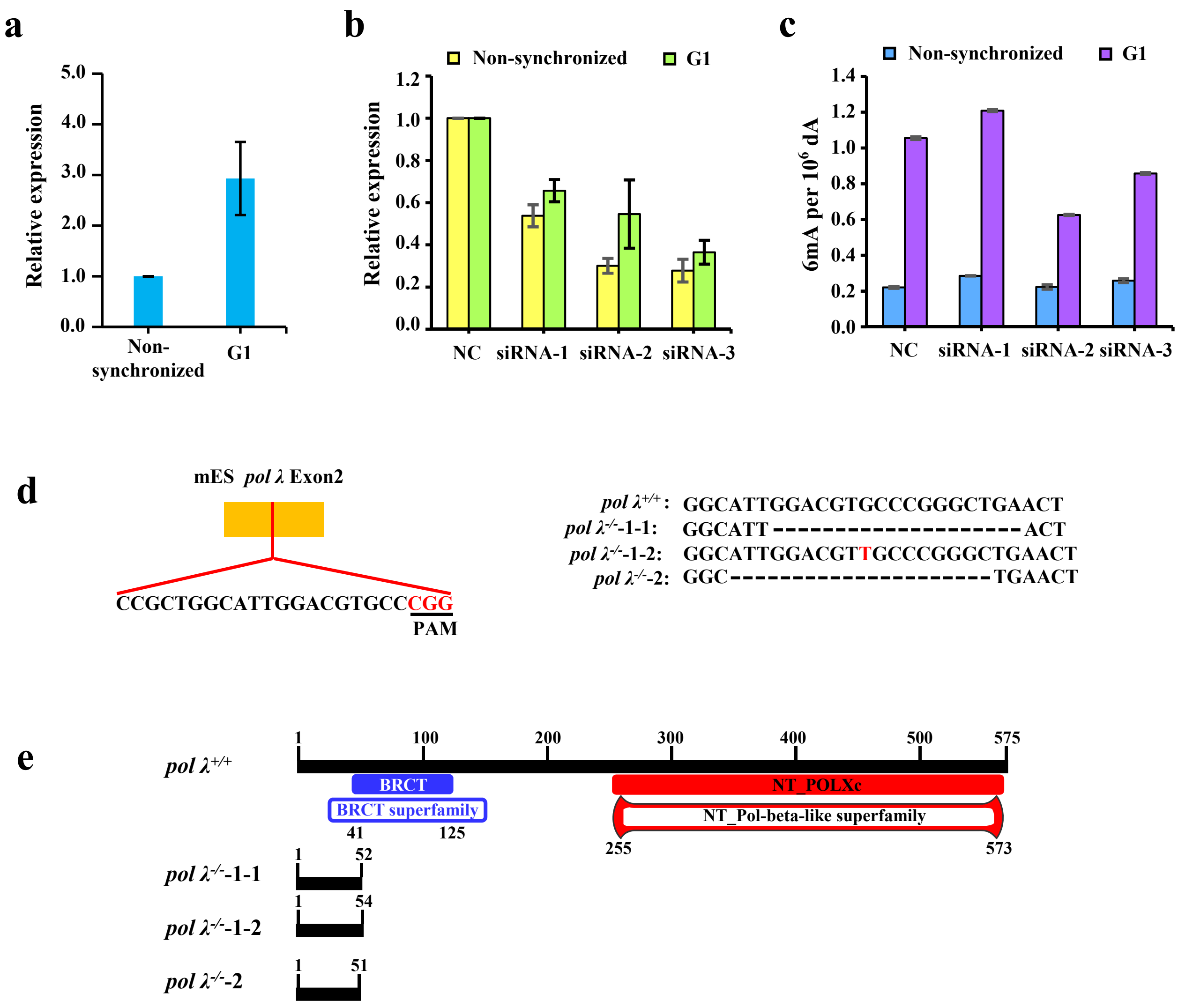

Fig. S8 Pol λ-mediated incorporation of 6mA in the genomes of mES cells at late G1 phase.

(**a**) qPCR analysis of pol λ mRNA expression in mES cells. The mES cells were untreated or treated with late G1-arresting L-mimosine. Error bars: s.d. n = 3. (**b**) qPCR analysis of pol λ mRNA expression for evaluation of siRNA knockdown. Error bars: s.d. n = 3. (**c**) UHPLC-MS/MS quantification of genomic 6mA level in pol λ-knockdown mES cells. Error bars: s.d. n = 3. (**d, e**) Schematic illustration of pol λ mutant allele gene using the CRISPR/Cas9 genome editing tool in mES cells. The sgRNA sequence and knockout sites (**c**) and the representative mutant pol λ protein sequences (**d**) were indicated. Note, mES cells at late G1 phase were obtained by the L-mimosine treatment.

a

pol λ$^{+/+}$

b

pol λ$^{-/-}$**-1**

c

pol λ$^{-/-}$**-2**

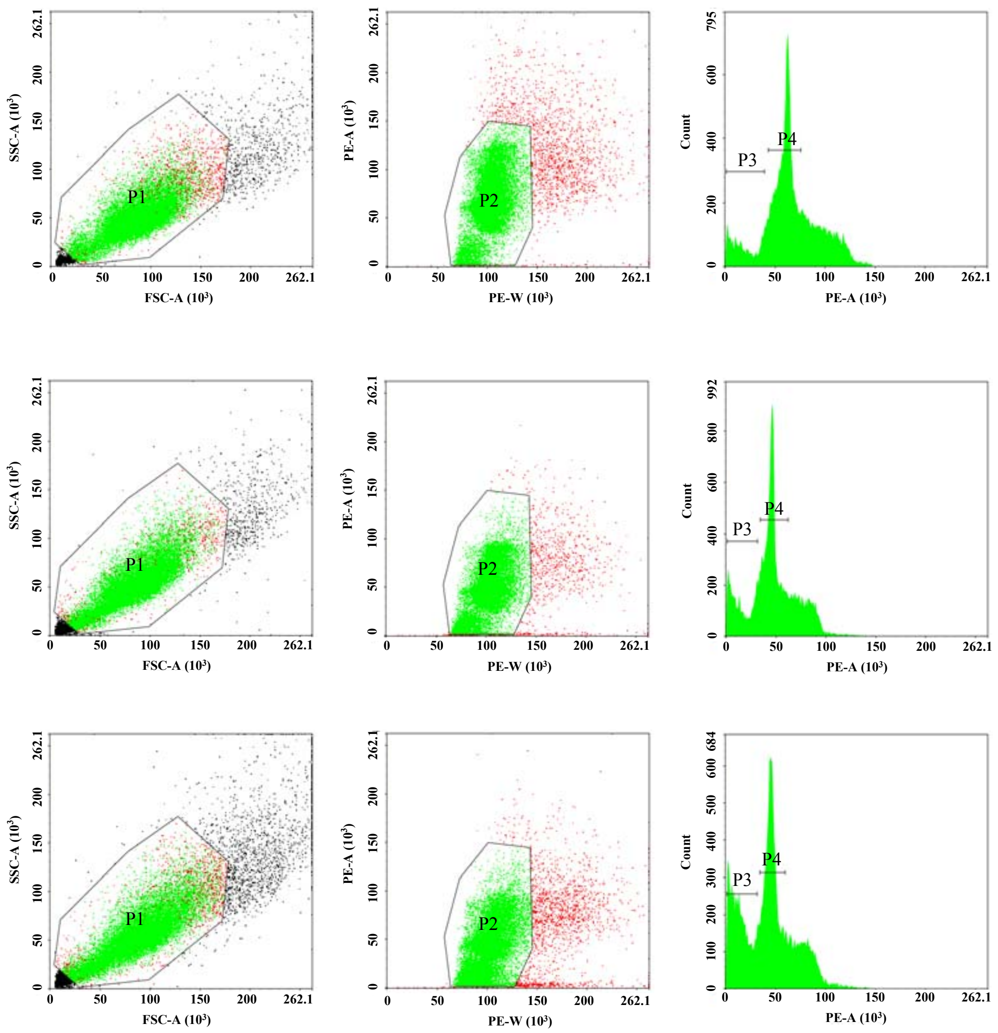

d

Population	%Total (*pol λ*$^{+/+}$)	%Total (*pol λ*$^{-/-}$-1)	%Total (*pol λ*$^{-/-}$-2)
P1	91.6	88.8	85.1
P2	83.4	83.7	76.6
P3	11.4	25.0	26.7
P4	40.5	29.7	29.7

Fig. S9 Flow cytometry for cell sorting and apoptosis analysis

(a-c) Flow cytometry sorting of *pol λ*$^{-/-}$ mES cells at the sub G1 and G1 phases. The cells were stained by propidium iodide (PI) for sorting. P1: The total cells except debris and nuclei; P2: the single cells; P3: the cells at the sub G1 phase; P4: the cells at the G1 phase. (**a**), *pol λ*$^{+/+}$; (**b**), *pol λ*$^{-/-}$-1; (C), *pol λ*$^{-/-}$-2. (**d**) The percentage of P1 - P4 accounted for the total cells. The presence of the cells at the sub-G1 phase indicates the occurrence of apoptosis.

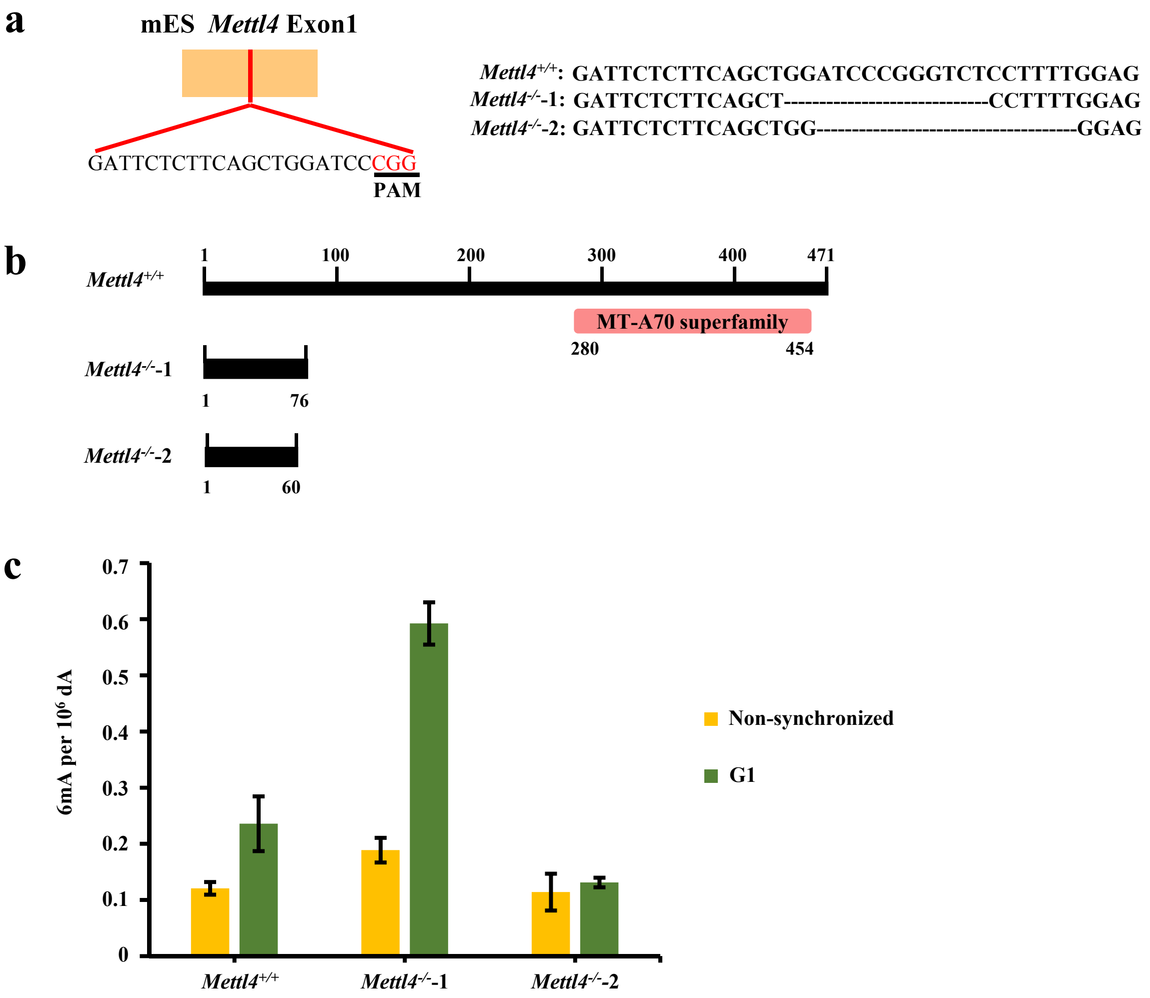

Fig. S10 Effect of *Mettl4* on genomic 6mA in mES cells.

(**a**) Schematic representation of *Mettl4* mutant allele genes using CRISPR-Cas9 system. The sgRNA sequence and knockout sites are indicated. (**b**) The representative mutant *Mettl4* protein sequences are indicated. (**c**) UHPLC-MS/MS quantification of the 6mA levels in *Mettl4*$^{+/+}$ and *Mettl4*$^{-/-}$ mES cells. Error bars: s.d. n = 3.

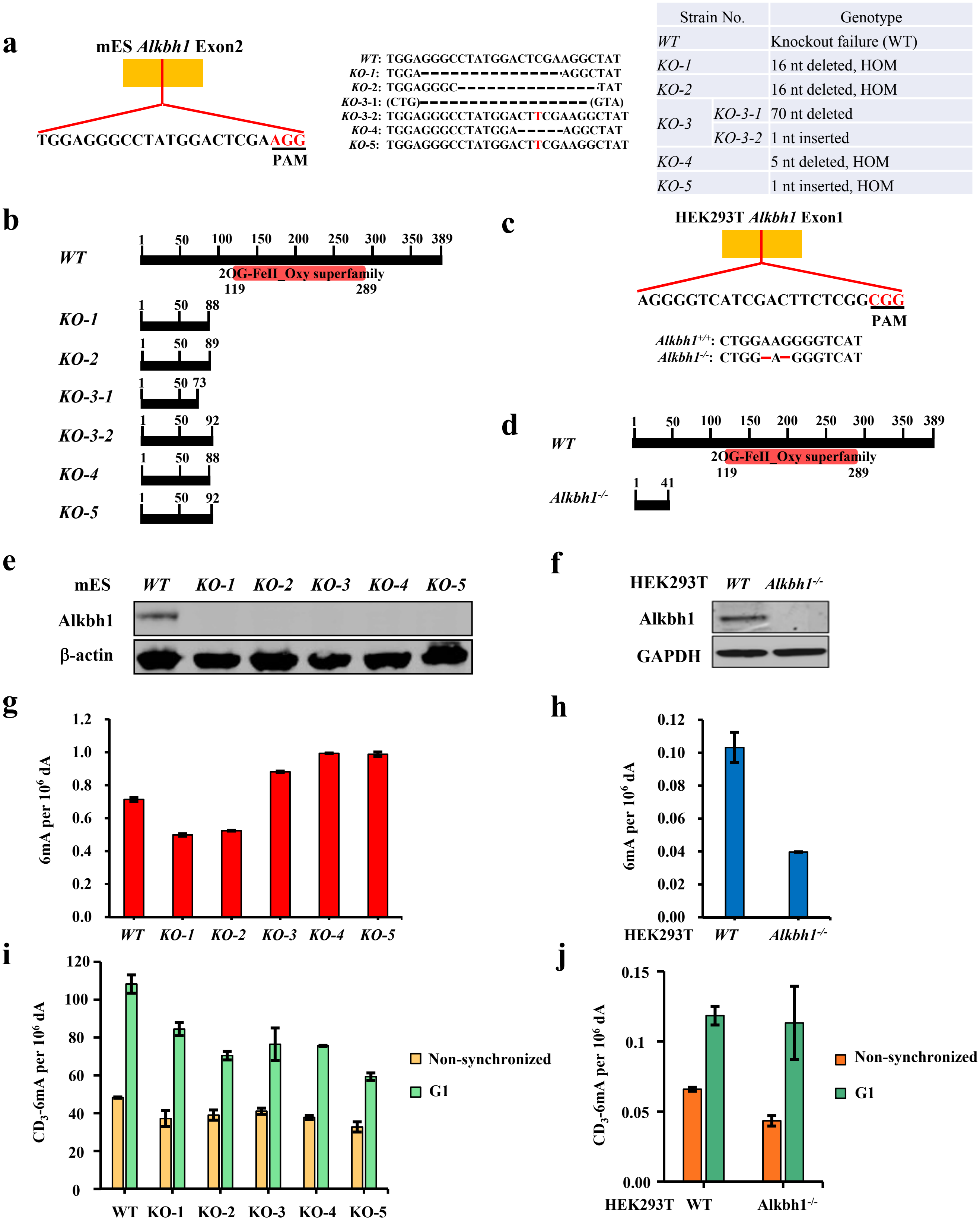

Fig. S11 Effect of *Alkbh1* on genomic incorporation of 6mA in mES and HEK293T cells. (**a, c**) The strategies were designed for in vivo CRISPR/Cas9-mediated *Alkbh1* gene in mES cells and HEK293T cells, respectively. The sgRNA sequence and knockout sites were indicated. (**b, d**) Schematic illustration of the mutant *Alkbh1* protein information. (**e, f**) Western blot analysis of *Alkbh1* knockout in mES cells (**e**) and HEK293T (**f**) cells, respectively. (**g, h**) UHPLC-MS/MS quantification of genomic 6mA levels in mES cells (**g**) and HEK293T cells (**h**). (**i, j**) UHPLC-MS/MS quantification of genomic [CD_3]-6mA in mES (**i**) and HEK293T cells (**J**) while the two cells were treated with 10 μM [CD_3]-m^6A, respectively. Error bars: s.d. n = 3.
